# Capability wellbeing of parents caring for children with rare diseases: a national cross-sectional study

**DOI:** 10.1186/s12889-025-25452-8

**Published:** 2025-11-28

**Authors:** Jiazhou Yu, Huanyu Zhang, Shanquan Chen, Richard H. Xu, Shuyang Zhang, Dong Dong

**Affiliations:** 1 JC School of Public Health and Primary Care, The Chinese University of Hong Kong, Shatin, Hong Kong SAR, China; 2 Clinical Big Data Research Center, The Seventh Affiliated Hospital, Sun Yat-Sen University, Shenzhen, 518107 China; 3 Shenzhen Research Institute, The Chinese University of Hong Kong, Shenzhen, China; 4 School of Public Health, The University of Hong Kong, Hong Kong, Hong Kong SAR, China; 5 Department of Rehabilitation Sciences, The Hong Kong Polytechnic University, Hong Kong SAR, China; 6 Department of Cardiology, Peking Union Medical College and Chinese Academy of Medical Science, Beijing, China

**Keywords:** Children, Rare diseases, Parental wellbeing, Caregiver, China

## Abstract

**Background:**

Rare diseases impose significant burden on families, particularly parents, of affected children, involving intensive caregiving, financial hardship, and social isolation. However, there is a scarcity of research exploring the broader dimensions of parental wellbeing, such as enjoyment and achievement. This study aims to assess the capability wellbeing among parents caring for children with rare diseases and to identify the contributing factors of parental wellbeing.

**Methods:**

A nationwide cross-sectional survey was conducted on 10,207 parents of children diagnosed with 28 different rare diseases in China in 2019–2020. The survey collected disease-related factors of the children and socio-demographic and psychosocial factors of parents. The capability wellbeing of parents was measured using the ICEpop CAPability Instrument for Adults (ICECAP-A), with a score ranging from 0 (no capability) to 1 (full capability). Multivariable linear regression was employed to identify factors associated with parental capability wellbeing.

**Results:**

Parents demonstrated impaired capability wellbeing (ICECAP-A mean score: 0.64), particularly in abilities related to stability, achievement, and autonomy. Mothers (ICECAP-A mean score 0.63) reported poorer wellbeing than fathers (ICECAP-A mean score 0.64). Severe disease condition, lack of treatment, diagnostic delay in children, low socio-economic status, lack of social support, financial burden, and work disruption among parents were found to undermine parental capability wellbeing.

**Conclusion:**

Parents caring for children with rare diseases face multifaceted challenges that negatively affect their overall capability wellbeing, particularly in areas of stability, achievement, and autonomy. Gender-specific challenges call for tailored services and support for mothers and fathers.

**Supplementary Information:**

The online version contains supplementary material available at 10.1186/s12889-025-25452-8.

## Background

Rare diseases (RDs) refer to health conditions that affect a small number of people compared to more common diseases within a population [1]. They are defined as conditions affecting no more than 5 in 10,000 people in the European Union (EU) [2] and as conditions affecting fewer than 200,000 individuals in the United States [3]. Examples of RDs include spinal muscular atrophy, cystic fibrosis, and amyotrophic lateral sclerosis. Although individually rare, RDs collectively affect an approximately 350 million people worldwide [4]. RDs are associated with mortality, morbidity, and disability [5]. Regional studies on specific conditions have reported that patients with RDs face more than double the mortality rate of the general population, alongside increased risks of complications and reduced life expectancy [6, 7]. There are more than 6,400 identified unique RDs, with about 80% of genetic origin and at least half of all RDs beginning in childhood [8]. In a national study from Ireland, RDs contributed to 58.6% of pediatric mortality in children aged 0–14 years [9]. Often genetically based and debilitating, RDs exert substantial negative impacts not only on the children but also on their families and caregivers [10].

In China, an estimated 16.8 million individuals, including 11.8 million children, are currently living with RDs [11]. While China has not yet passed any legislation on RDs or orphan drugs, several policies and national strategies have been introduced since 2015, such as publishing China’s National Lists of Rare Diseases and prioritizing new drugs to treat RDs and children for review and approval [12]. Despite ongoing efforts, China’s health and social policies for RDs still fall behind the United States and European Union [13, 14]. In a nationwide study in China, pediatric patients with RDs experience impaired quality of life across physical, emotional, and social functioning, with burden varying by condition and disease severity [15].

Parents of children with RDs face challenges that may affect their overall capability to lead fulfilling lives, for example, long-term caregiving, diagnostic delays, limited access to information and support, high treatment costs, and the need to adapt work patterns and social participation [16–19]. Studies have documented lower quality of life and greater psychological distress in parents caring for children with RDs, compared to parents of healthy children or children with more common chronic conditions [19, 20]. In China, traditional Confucian values continue to influence modern social norms, including gender expectations. Women are typically viewed as primarily responsible for domestic care work, while men serve as economic provider [21]. As a result, Chinese mothers are often more willing to sacrifice their personal needs to fulfill familial responsibilities [22]. These gender-based expectations may become more prominent when intensive caregiving obligations are involved. It is reported that in a family, fathers and mothers of children with RDs face distinct challenges. Typically, mothers report a lower quality of life [20], often due to career adjustments and shifts in family dynamics, while fathers more frequently confront economic challenges [23]. As the primary caregivers and supporters of children with RDs, parents’ wellbeing and capability are crucial in improving pediatric patient outcomes. Traditional health-related quality of life (HRQoL) measures, focusing predominantly on health status, often fail to capture the entire spectrum of challenges these parents face, leaving out the broader aspects of wellbeing such as enjoyment and achievement [20, 23].

In this context, the Capability Approach, as proposed by Amartya Sen, has emerged as a significant approach in measuring overall individual wellbeing [24–26]. Offering an alternative to traditional HRQoL measures, the ICEpop CAPability (ICECAP) instrument was developed to assess capability wellbeing and has been increasingly used in economic evaluations [27]. Grounded in Sen’s framework, ICECAP measures a range of capabilities, focusing on an individual’s broader wellbeing and their ability to engage in meaningful life activities, rather than merely their health status [28]. Complementing Sen's framework, Nussbaum’s elaboration of central human capabilities emphasizes how structural and cultural constraints, particularly gendered caregiving expectations, can limit individuals’ freedom to realize these valued activities [29]. From this perspective, wellbeing must be understood not only as the presence of health or resources but also as the opportunity to achieve dignity, autonomy, and affiliation. This integrated approach provides a more comprehensive picture of individual wellbeing than HRQoL measures and has been increasingly applied in healthcare resource allocation decisions [30–32].

Given the multifaceted challenges faced by parents of children with RD and the limitation of traditional HRQoL measures, it is critical to apply the Capability Approach when investigating the parental wellbeing. Applying this approach would provide a more nuanced understanding of their experiences, revealing not only the health-related aspects but also their ability to achieve personal goals, maintain social ties, and enjoy life despite their caregiving responsibilities. This perspective is essential for developing targeted interventions and support systems that address the unique and diverse needs of parents caring for children with RDs, which in turn benefits their overall quality of life and ability in caregiving.

Through a nationwide survey in China, this study aims to gain a detailed understanding of the capability wellbeing among parents of children with RDs, with a particular focus on gender differences. This study also seeks to identify both disease-related and psychosocial factors that contribute to the capability wellbeing of parents. By providing a comprehensive overview of parental wellbeing and revealing gender-specific experiences, the study aims to provide evidence for specialized, tailored support measures to mitigate the adverse effects of caring for children with RDs and to meet the diverse needs of both fathers and mothers in this vulnerable population.

## Methods

### Study design and data collection

This study uses data from a nationwide cross-sectional survey of RD patients in China, conducted under the collaboration among China Alliance for Rare Disease, Peking Union Medical College Hospital, the Illness Challenge Foundation, and the Chinese University of Hong Kong. The data collection was conducted between August 2019 and January 2020. The details of study design and data collection process of the original study have been described elsewhere [15, 33].

Briefly, data were collected through an online questionnaire. Two versions of questionnaire (self-report and proxy-report) were designed. The questionnaires collected information of the patient, with the same measures but with questions differently phrased to generate more accurate responses, including: (1) patient’s sociodemographic background information (e.g., age, sex, educational attainment, and income), (2) patient’s diagnosis and treatment (e.g., symptom onset and diagnosis, and current treatment status). The proxy-reported version of questionnaire included an additional section that collects information of the proxy (i.e., caregiver), including: (1) caregiver’s background information (e.g., relationship with the patient, sex, marital status, education); (2) caregiver’s socio-psychological information (e.g., social support, perceived impact on work).

The survey started with questions for identifying the target responses, including age and identity (patient or caregiver of the patient). Respondents younger than 18 years were required to end the survey and instructed to pass the survey to their parents or legal guardians. Each participant was automatically directed to either the self-reporting (filled by the patient) or proxy-reporting (filled by the caregiver) version based on their choice of self-identity. The research team provided real-time online support to rare disease patient organizations (RDPOs) during the survey period. Collected answers were manually checked by researchers, and extreme and unusual answers were manually verified.

As the survey was conducted on an online platform, electronic informed consent was obtained from all participants through a mandatory process where participants were required to read the consent form and actively click on “consent to participate” before entering the study. If they clicked on “not consent to participate”, the survey would end automatically. For participants under the age of 18, electronic informed consent was obtained from their parents or legal guardians using the same process.

### Participants

Due to the lack of sampling frame for rare disease patients in China, the patients in the original dataset were recruited using a non-probability sampling method. In 2018, China released the First National List of Rare Diseases, which included 121 different conditions [34]. By 2019, there were established national RDPOs for 56 of these 121 conditions. After inviting all available RDPOs to join the study, 32 RDPOs representing 33 diseases agreed to participate. Participants of this study were recruited through the 32 national RDPOs that represented patients with 33 RDs from China’s first official list of RDs. The patients were considered eligible to join the study only after RDPOs verified their diagnosis records. The survey collected a total of 20,804 responses.

The current study focuses on the parental wellbeing of pediatric RD patients. Therefore, parents of pediatric RD patients, who provided proxy-reports in the survey, were considered the study participants of this study. These proxy reports included information about both the child and the parent themselves. In this analysis, responses from the original survey were excluded if they are: (1) self-report or proxy-report for a patient aged at ≥ 18 (*n* = 7,424); (2) proxy-report by a caregiver other than a parent (*n* = 1,695); (3) incomplete responses on key variables (*n* = 1,478). Finally, 10,207 parents of pediatric RD patients were included (Supplementary Fig. 1).

### Instruments

#### Capability wellbeing

The participant’s (parent’s) capability wellbeing was measured by the ICECAP-A, which consists of five attributes, each indicating a certain ability: (1) stability: to feel settled and secure; (2) attachment: to have love, friendship, and support; (3) autonomy: to be independent; (4) achievement: to achieve and progress in life; (5) enjoyment: to experience enjoyment and pleasure. Individuals are asked to select the level of each of the five capability attributes (from four-level options where Level 4 indicates full capability and Level 1 indicates no capability). The instrument has been adapted and validated in the Chinese population [35]. In developing the Chinese version of the ICECAP-A, researchers made slight modifications to the questionnaire’s wording to ensure cultural relevance and ease of understanding in the Chinese context. Specifically, the term “love/friendship” was replaced with “kindness”, and “feel settled” was changed to “stability”. Additionally, a brief introduction was added to clarify that the concept of capability pertains to the respondent’s health, work, family economic conditions, and social relationships [35]. In this study, due to the lack of tariff specific to the Chinese population, we calculated the utility score of ICECAP-A with best–worst scaling using tariffs obtained from the UK general population (Supplementary Table 1) [36]. We first obtained a utility score for each individual attribute based on item-specific tariff, then calculated a total score by summing the values across the individual attributes [36]. The total utility score ranges between 0–1, indicating the capability wellbeing ranged between no capability and full capability. In this analysis, based on the responses’ indication of capability level, we categorized response at Level 3–4 as no/mild impairment, while response at Level 1–2 as moderate/severe impairment in capability in each attribute.

#### Child and parental variables

The child’s background information was proxy-reported by the parents, including the child’s age, sex, household registration (rural, urban or overseas), dependence on assistive devices (none, moderate, substantial), active treatment defined by the latest treatment within six months prior to the survey (yes, no), recent diagnosis defined by the definite diagnosis within one year prior to the survey (yes, no), diagnostic delay defined as the number of years between symptom onset and definite diagnosis (< 1 year, 1–2 years, ≥ 3 years), and comorbidity with other rare diseases (yes, no). The patients were diagnosed with 28 different RDs which were grouped into four categories according to the 10th version of the International Classification of Diseases and Related Health Problems (ICD-10): endocrine, nutritional and metabolic diseases; diseases of the nervous system; congenital malformations, deformations; others. The parent’s information included sex, role in caregiving (primary, non-primary), marital status (married, divorced/separated/widowed), educational attainment (below high school, high school to below university, university or above), employment (yes, no), annual household income (< ¥50,000, ¥50,000–100,000, > ¥100,000) (CNY 1 = USD 0.14). We examined the parent’s knowledge of disease using three questions related to basic knowledge of the specific disease, where one point was given for correct response and zero for incorrect response. A summary score ranging from 0–3 was generated, with higher score indicating higher knowledge of disease. Social support was evaluated by the Chinese version of the 8-item Medical Outcomes Study Social Support Survey (mMOS-SSS) [37]. The total score ranges between 0–100, with a higher score indicating a higher level of social support. We additionally asked parents how often they felt their work had been disrupted because of the child’s condition, examined by a 5-point Likert scale of “never”, “rarely”, “sometimes”, “often”, “always”. They were also asked how they perceived the child’s condition had burdened the household financial status, with 5-level responses of “none”, “mild”, “moderate”, “severe”, “very severe”. Severe work disruption was defined as occurring with a frequency of “often” to “always”.

### Statistical analysis

The background characteristics of children and parents were described as number and percentage for categorical variables and as mean and standard deviation (SD) for continuous variables. ICECAP-A score was presented as mean and SD across different background categories and compared using ANOVA. The responses to the five different attributes of ICECAP-A were summarized separately for mothers and fathers.

To identify the factors contributing to parent’s capability wellbeing, we conducted linear regression using ICECAP-A utility score separately for mothers and fathers. All variables had <1% missing data, except parental employment (2.6%), which were left as they were. Explanatory variables included children’s age, sex, household registration, dependence on assistive devices, active treatment, recent diagnosis, diagnostic delay, disease category and parents’ caregiving role, education attainment, employment, annual household income, knowledge of disease, social support level, and perceived impact on work and family financial burden. Univariable regression was first conducted, and variables significant at *p* < 0.10 level were further included in multivariable regression models. To further explore the contribution of factors on each attribute of capability wellbeing, we also conducted regression models with significant factors from multivariable models as predictors and five attribute-specific scores as outcomes. Coefficients (β) with 95% confidence interval (CI) were reported. The significance level was set at *p* < 0.05. The statistical analyses were conducted using Stata 16.0 (Stata Corp, College Station, TX).

The ethical approval of this study was obtained from the Survey and Behavioural Research Ethics Committee of the Chinese University of Hong Kong and the Ethical Committee of Peking Union Medical College Hospital.

## Results

### Basic characteristics

A total of 10,207 parents of pediatric patients with 28 different RDs were included in the study. The basic characteristics of children and parents are summarized in Table 1. The mean age of the pediatric patients was 5.45 ± 4.03 years. The most common diseases were endocrine, nutritional and metabolic diseases (43.6%) and diseases of nervous system (26.6%). More than half of the participants were mothers (72.9%). Mothers (75.5%) were more likely to be primary caregivers of the child compared to fathers (18.9%).

**Table 1:** Descriptive characteristics of pediatric patients and parents (*n* = 10,207) and ICECAP-A index score

Variable	n (%)	ICECAP-A index score	
		*Mean (SD)*	*p*^*1*^
Total sample	10207 (100%)	0.64 (0.22)	
**Child variables**			
Age			0.03
0–2	2892 (28.3%)	0.64 (0.23)	
3–7	4526 (44.3%)	0.63 (0.22)	
8–12	2070 (20.3%)	0.64 (0.23)	
13–18	719 (7.0%)	0.65 (0.22)	
Sex			0.004
Male	6403 (62.7%)	0.63 (0.23)	
Female	3804 (37.3%)	0.65 (0.22)	
Household registration			< 0.001
Rural	5590 (54.8%)	0.61 (0.23)	
Urban or overseas	4617 (45.2%)	0.67 (0.21)	
Dependence on assistive devices			< 0.001
None	6778 (66.4%)	0.67 (0.22)	
Moderate	1889 (18.5%)	0.59 (0.22)	
Substantial	1539 (15.1%)	0.57 (0.23)	
Active treatment			< 0.001
Non-active	2894 (28.4%)	0.62 (0.23)	
Active	7313 (71.7%)	0.64 (0.22)	
Diagnostic delay			< 0.001
< 1 year	6254 (73.1%)	0.64 (0.23)	
1–2 years	1733 (20.3%)	0.60 (0.23)	
≥ 3 years	573 (6.7%)	0.61 (0.22)	
Recent diagnosis			< 0.001
No	7051 (69.4%)	0.64 (0.22)	
Yes	3112 (30.6%)	0.62 (0.23)	
Category of disease			< 0.001
Endocrine, nutritional and metabolic diseases	4446 (43.6%)	0.66 (0.22)	
Diseases of the nervous system	2710 (26.6%)	0.59 (0.23)	
Congenital malformations, deformations and chromosomal abnormalities	1989 (19.5%)	0.62 (0.22)	
Others	1062 (10.4%)	0.69 (0.21)	
Comorbid rare disease			
No	9325 (91.4%)	0.65 (0.22)	< 0.001
Yes	882 (8.6%)	0.55 (0.24)	
**Parental variables**			
Sex			0.007
Male	2762 (27.1%)	0.65 (0.23)	
Female	7445 (72.9%)	0.63 (0.22)	
Primary caretaker of patient			0.0004
No	4093 (40.1%)	0.65 (0.22)	
Yes	6114 (59.9%)	0.63 (0.23)	
Marital status			< 0.001
Married	9192 (90.1%)	0.64 (0.22)	
Divorced/Separated/Widowed	1015 (9.9%)	0.59 (0.23)	
Educational attainment			< 0.001
Up to lower secondary	3612 (35.4%)	0.58 (0.24)	
Upper secondary	4204 (41.2%)	0.65 (0.21)	
Tertiary	2388 (23.4%)	0.70 (0.20)	
Employment			< 0.001
Unemployed	4272 (43.0%)	0.59 (0.23)	
Employed	5674 (57.1%)	0.67 (0.21)	
Annual household income			< 0.001
< ¥50,000	4345 (42.6%)	0.58 (0.23)	
¥50,000–100,000	4010 (39.3%)	0.65 (0.21)	
> ¥100,000	1846 (18.1%)	0.73 (0.19)	
Social support, mean (SD)	40.22 (20.33)	——	——
Perceived financial burden of the condition			< 0.001
None to moderate	4206 (41.2%)	0.73 (0.18)	
Severe	6001 (58.8%)	0.57 (0.23)	
Perceived impact on work			< 0.001
Never to sometimes	3540 (53.1%)	0.73 (0.18)	
Often or always	3128 (46.9%)	0.58 (0.22)	

*ICECAP-A* ICEpop CAPability Instrument for Adults, *SD* standard deviation, —— Not applicable. CNY 1 = USD 0.14

^1^*P*-value of ANOVA test, *p* < 0.05 indicates statistically significant differences across socio-demographic categories

### Capability wellbeing

The average utility score of ICECAP-A was 0.64 ± 0.22 among all participants, 0.65 ± 0.23 among fathers, and 0.63 ± 0.22 among mothers. The score was found significantly different by all the variables (Table 1). By 28 different disease (Supplementary Table 2), the mean score was the highest among parents of children with albinism (0.75 ± 0.22), systemic sclerosis (0.73 ± 0.20), and congenital adrenal hyperplasia (CAH) (0.72 ± 0.20) whereas the lowest scores were observed among parents of children with idiopathic pulmonary arterial hypertension (IPAH) (0.48 ± 0.24), Huntington’s disease (0.52 ± 0.24), and Dravet syndrome (0.53 ± 0.23). Mothers generally showed similar or lower scores than fathers. Mothers of children with Kallmann Syndrome (0.41 vs 0.76), multiple sclerosis (0.61 vs 0.75), Neuromyelitis optica spectrum disorder (NMOSD) (0.65 vs 0.75), Huntington’s disease (0.42 vs 0.58) reported considerably lower scores (Supplementary Table 2).

**Fig. 1:**
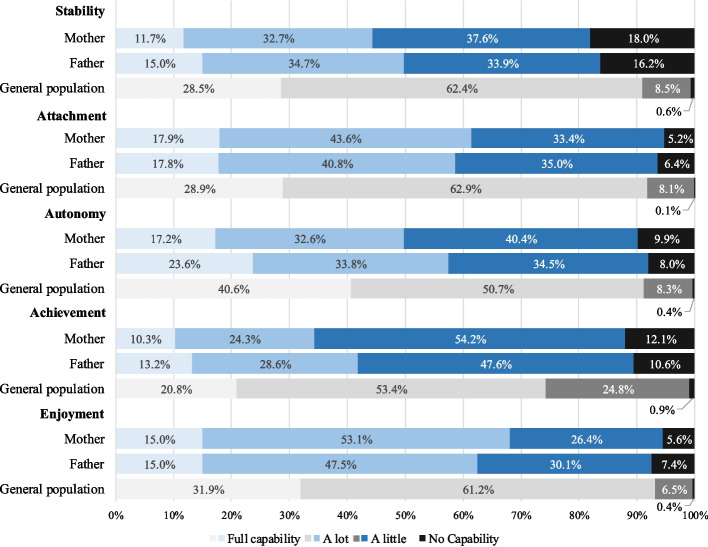
Distribution of responses on five attributes of ICECAP-A by role of participants, compared with general Chinese population measured by Tang et al., 2018 [35]

By attributes (Fig. 1), more than half of parents recorded the two lowest responses indicating moderate/severe capability impairment for achievement (65.3% of mothers, 58.2% of fathers) and stability (55.6%, 50.1%). On the other hand, approximately or over half of parents reported no/mild impairment on enjoyment (68.1%, 62.6%), attachment (61.5%, 58.6%), and autonomy (49.8%, 57.4%). The impairment was severe compared to health general populations in China [35] (Fig. 1). Compared to fathers, mothers generally demonstrated better capability in attachment and enjoyment but worse in stability, autonomy and achievement.

### Factors associated with capability wellbeing

The results of multivariable regression models are described in Table 2. When using ICECAP-A score as a continuous outcome, higher scores were observed in mothers of older children (β = 0.002, 95% CI 0, 0.003) and children under active treatment (β = 0.02, 95% CI 0.01, 0.03). Mothers who were primary caregiver also showed higher scores (β = 0.02, 95% CI 0.01, 0.03). Higher education (upper secondary: β = 0.03, 95% CI 0.02, 0.05; tertiary: β = 0.04, 95% CI 0.02, 0.06), higher income (middle: β = 0.03, 95% CI 0.02, 0.04; high: β = 0.05, 95% CI 0.03, 0.07), and greater social support (β = 0.003, 95% CI 0.002, 0.003) were positively associated with scores. Conversely, factors negatively associated with mothers’ ICECAP-A scores included children’s dependence on assistive devices (moderate: β = −0.03, 95% CI −0.05, −0.01; substantial: β = −0.04, 95% CI −0.06, −0.03), severe perceived impact on work (β = −0.09, 95% CI −0.10, −0.07) and family financial burden (β = −0.07, 95% CI −0.08, −0.06). Compared to mothers of children with endocrine, nutritional and metabolic disease, those of children with diseases of nervous system (β = −0.06, 95% CI −0.07, −0.04) and congenital malformations, deformations (β = −0.02, 95% CI −0.04, −0.01) demonstrated significantly lower score.

**Table 2:** Multivariable regression to identify factor associated with ICECAP-A index score by role of respondents

Variable	ICECAP-A index score	
	Mother	Father
**Child variables**	*Coeff (95% CI)*	*Coeff (95% CI)*
Age	0.002 (0, 0.003)*	——
Dependence on assistive devices		
None	Ref	Ref
Moderate	−0.03 (−0.05, −0.01)***	−0.04 (−0.06, −0.02)***
Substantial	−0.04 (−0.06, −0.03)***	−0.06 (−0.08, −0.04)***
Active treatment		
Non-active	Ref	Ref
Active	0.02 (0.01, 0.03)**	0.02 (0.00, 0.04)*
Diagnostic delay		
< 1 year	——	Ref
1–2 years	——	−0.02 (−0.04, 0.00)*
≥ 3 years	——	−0.01 (−0.04, 0.03)
Disease category		
Endocrine, nutritional and metabolic diseases	Ref	Ref
Diseases of the nervous system	−0.06 (−0.07, −0.04)***	−0.03 (−0.05, 0.00)*
Congenital malformations, deformations	−0.02 (−0.04, −0.01)**	0.01 (-0.04, 0.00)
Others	0.01 (−0.01, 0.03)	0.04 (0.01, 0.07)**
**Parental variables**		
Primary caregiver of the patient		
No	Ref	——
Yes	0.02 (0.01, 0.03)***	——
Educational attainment		
Up to lower secondary	Ref	Ref
Upper secondary	0.03 (0.02, 0.05)***	0.04 (0.02, 0.06)***
Tertiary	0.04 (0.02, 0.06)***	0.06 (0.03, 0.08)***
Employment		
Unemployed	——	Ref
Employed	——	0.03 (0.00, 0.05)*
Annual household income		
Low (< ¥50,000)	Ref	Ref
Middle (¥50,000–100,000)	0.03 (0.02, 0.04)***	0.03 (0.01, 0.05)**
High (> ¥100,000)	0.05 (0.03, 0.07)***	0.03 (0.00, 0.06)*
Social support	0.003 (0.002, 0.003)***	0.003 (0.002, 0.003)***
Perceived financial burden of the disease		
None to moderate	Ref	Ref
Severe	−0.07 (−0.08, −0.06)***	−0.07 (−0.09, −0.05)***
Perceived work disruption due to the disease		
Never to sometimes	Ref	Ref
Often or always	−0.09 (−0.10, −0.07)***	−0.09 (−0.11, −0.07)***

*ICECAP-A* ICEpop CAPability Instrument for Adults, *Ref* reference group

^*^p < 0.05, ^**^p < 0.01, ^***^p < 0.001

CNY 1 = USD 0.14; —— not included in the final model due to non-significance. Children’s sex, rurality of household registration, recent diagnosis status, presence of comorbid rare disease, parents’ marital status, and parent’s knowledge of disease did not show any significant association with ICECAP-A score in multivariable models regardless of the respondent’s role

Among fathers, higher ICECAP-A scores were associated with having children under active treatment (β = 0.02, 95% CI 0.00, 0.04) and being employed (β = 0.03, 95% CI 0.00, 0.05). Education (secondary: β = 0.04, 95% CI 0.02, 0.06; tertiary: β = 0.06, 95% CI 0.03, 0.08), income (middle: β = 0.03, 95% CI 0.01, 0.05; high: β = 0.03, 95% CI 0.00, 0.06), and social support (β = 0.003, 95% CI 0.002, 0.003) were positively associated with score. Children’s dependence on assistive devices (moderate: β = −0.004, 95% CI −0.06, −0.02; substantial: β = −0.06, 95% CI −0.08, −0.04), diagnostic delay of 1–2 years for children (β = −0.02, 95% CI −0.04, 0.00), severe impact on work (β = −0.09, 95% CI −0.10, −0.07) and on financial burden (β = −0.07, 95% CI −0.09, −0.05) were associated with lower scores. Fathers of children with diseases of the nervous system had lower score than those of children with endocrine, nutritional and metabolic diseases (β = −0.03, 95% CI −0.05, 0.00). Children’s sex, rurality of household registration, recent diagnosis status, presence of any comorbid rare disease, parents’ marital status, and parent’s knowledge of disease did not show any significant association with ICECAP-A score regardless of the participant’s role.

**Fig. 2:**
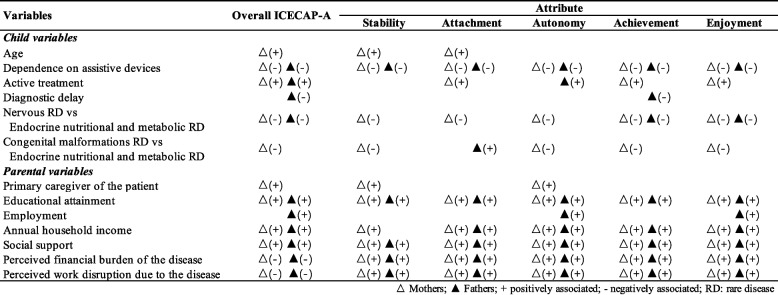
Significant associations between variables and overall ICECAP-A score and by attribute among mothers and fathers

Attribute-specific analysis revealed distinct patterns for both parents (Supplementary Table 2). For mothers, child’s age positively correlated with stability and attachment, while active treatment status enhanced attachment, achievement, and enjoyment. Primary caregivers showed improved stability and autonomy, and higher education was associated with all attributes except enjoyment. Child's device dependence, disease category, mother’s income, social support, and perceived burdens significantly affected all attributes. For fathers, active treatment enhanced autonomy, while diagnostic delays reduced achievement capability. Employment improved autonomy and enjoyment, and social support enhanced stability, attachment, achievement, and enjoyment. Device dependence, education level, and perceived financial and work impacts significantly influenced all paternal capability attributes. The significant associations found for ICECAP-A overall score and by attributes among mothers and fathers are summarized in Fig. 2.

## Discussion

Parents caring for children with RDs demonstrated impaired capability wellbeing (ICECAP-A utility score mean 0.64) compared to that of the general population in China (mean 0.85) [35]. Mothers reported poorer capability wellbeing than fathers, particularly in stability, autonomy, and achievement. Parents of children who were dependent on assistive devices, not under active treatment, with disease of nervous system, and parents with lower education, lower household income, higher perceived financial burden, and higher perceived work disruption showed significantly poorer capability wellbeing.

The parents of children with RDs experience significantly impaired capability wellbeing compared to the healthy population in China (mean 0.85) [35], Hong Kong (mean 0.85) [38], and European countries (mean 0.83–0.89) [36, 39, 40]. Although physically disease-free, the parents reported level of capability wellbeing comparable to individuals with depression (mean 0.63) [39], opiate dependence (mean 0.66) [28], and dermatological symptoms (mean 0.69) [41]. The level was also lower than patients with diabetes, arthritis, asthma, cancer, heart disease, and hearing loss (mean 0.79–0.85) [39]. By focusing on caregivers rather than patients, the findings provide a nuanced perspective on spillover effects of pediatric RDs, as evidenced by parents’ capability impairment comparable to individuals affected by own health conditions. By attributes, we found that the most noticeable impairment with stability and achievement, and attachment when compared to the healthy Chinese population [35]. This reflects that parents caring for children with RDs often experience a sense of uncertainty, instability, and lack of support. This is likely related to several factors: (1) the limited knowledge and expertise in treating RDs can lead to uncertainty in children’s prognosis; (2) the high caregiving demand and long-term dedication can consume substantial physical, emotional, and financial capacity. It thereby interferes with the parent’s personal or professional pursuits, hindering their ability to achieve progress in various areas of life; (3) the burden of caregiving may affect family relationships and social participation, which contributes to a feeling of isolation. These experiences have been previously documented among parents of children with rare diseases in a qualitative study from Italy [23]. Yet our quantitative approach further extends the evidence by measuring the magnitude of these effects across a large, diverse sample.

Previous studies show that mothers have lower mental wellbeing relative to fathers [42, 43] and face disproportionate caregiving burden [44]. This gender-specific impacts align with our findings, reflecting societal expectations and traditional gender roles [45]. In Chinese culture, women prioritize familial responsibilities more than men do [22]. This is supported by our finding that mothers were considerably more like to be the primary caregiver than fathers. Similarly, in Italy, the caregiving burden on mothers is also tied to traditional gender norms, but fathers’ roles appear less constrained by societal expectations compared to China, where familial responsibilities are strongly emphasized [23]. Our capability-based approach adds to the literature by exploring how these gender-specific expectations may impact individual capability domains differently in mothers and fathers. We found that compared to fathers, mothers reported more impaired capability wellbeing, particularly in stability, autonomy, and achievement. The demanding caregiving responsibilities can lead to career adjustment and personal sacrifices for mothers. Additionally, the debilitating nature of most RDs causes slow and uncertain health progress in children, which can undermine the sense of security and achievement in mothers. Fathers, on the other hand, were more impaired in attachment and enjoyment. This may be attributable to the predominant role of fathers as providers for their families. The financial strain related to the child’s treatment may pressure fathers to extend work or undertake additional job responsibilities. These responsibilities can thereby limit a father’s involvement in social and leisure activities. Furthermore, societal expectations regarding fathers’ role as financial providers may contribute to suppression of emotional expression and reduce their willingness to seek support. This is reflected in our findings that unemployment was identified as a significant contributor to fathers’ impaired wellbeing. Compared to Western contexts, the reinforced societal expectations placed on fathers to fulfill familial financial responsibilities in China may result in higher impairment on paternal wellbeing. These cultural differences necessitate tailored interventions that address the unique burdens faced by parents in different cultural contexts.

Our findings on the negative impact of children’s disease severity, lower socio-economic status, and insufficient social support were consistent with the existing evidence on parental QoL [42, 46–49]. Previous evidence has shown that children’s age was negatively associated with general parental QoL [44]. In contrast, we found that the mother’s capability wellbeing increases with the child’s age, mainly attributable to increased ability to feel settled and to have love and support. This difference likely reflects the unique focus of capability measures on freedom of fulfilling meaningful activities [28], unlike traditional QoL assessments that emphasize immediate functioning and satisfaction [50]. The observed positive association with child’s age may be contributed by adaptation processes, such as increased caregiving experience, knowledge, and improved coping strategies, which tended to be overlooked by traditional QoL measures. We noted that children’s active treatment status and timely diagnosis were associated with better parental wellbeing, particularly affecting the capability in achievement. This reflects that treatment and diagnosis progress may boost parents’ confidence and hope. The perceived financial strain and work disruption both associated with lower capability wellbeing. This indicates that parents encounter complex challenges in seeking a balance between caregiving roles, economic endeavors, and occupational obligations.

Although the included parents in our study were caring for children diagnosed with diverse RDs, the impairment in parental capability wellbeing was universally observed across all disease groups. Previous studies have found that parents of children with diseases of more severe form demonstrated lower QoL than those of children with a milder form [51, 52]. A previous study of Chinese pediatric patients with rare diseases reported that conditions causing neuromuscular symptoms, such as Dravet syndrome, demonstrated high health impact and disease-related expenses [15]. In our study, we found that neurological disorders such as Dravet syndrome impair parental wellbeing more than endocrine, nutritional, and metabolic diseases. This disparity likely reflects the greater caregiving burden, economic strain, and uncertainty surrounding prognosis, all of which are particularly pronounced in neurological disorders [15]. Such conditions often require constant supervision, frequent medical interventions, and emergency care, which can incur psychological distress and disrupt parents’ professional and personal lives [19, 53, 54]. For example, Dravet syndrome is characterized by severe epilepsy, developmental delays, and high seizure frequency, often leading to substantial caregiving demands. A qualitative study on Dravet syndrome reported that the demands of constant supervision can negatively affect caregivers’ social life, finances, and family interactions [55], for example, one parent noted “*I’ve had a hard time with work. I have changed job, been fired…because I had to go in by ambulance with him and so many other things*”, which support our finding on lower sense of stability and achievement; another noted “*We can almost never do anything else like other people… There is always someone who has to be able to drive or keep track of the situation*”, which reflects reduced autonomy and enjoyment [55]. In contrast, in our study, parents of children with diseases such as albinism reported higher ICECAP-A scores, possibly due to the relatively lower caregiving demands and less immediate health-related risks associated with these conditions. Albinism, while associated with visual impairment and potential social stigma, typically does not require the type of intensive, day-to-day medical management seen in neurological disorders. This highlights how disease-specific factors, such as severity, prognosis, caregiving requirements, and the risk and frequency of acute episodes, can contribute to variations in parental wellbeing across different RDs. Therefore, there is a need for targeted social support interventions, including respite services and workplace arrangements, particularly for parents of children with high-burden diseases like neurological disorders.

This study is the first to explore the capability wellbeing among parents caring for children with RDs. It employs the capability framework which considers broader life aspects such as enjoyment and social participation, rather than focusing only on health status, providing a more comprehensive understanding of parental experience and the spillover effect of RDs. Another notable strength is the large sample size collected from a nationwide survey. Additionally, the stratified analysis allowed for the identification of distinct challenges faced by mothers and fathers, which can facilitate formulation of tailored measures.

There are several limitations to this study. First, the data were self-reported by participants and may involve reporting bias. To reduce this bias, we used a web-based survey, which generates more accurate outcomes than telephone surveys, particularly with relatively sensitive topics [56]. Patients were reassured of anonymity to reduce social desirability bias. Second, the cross-sectional design precluded establishment of causal relationship between the examined factors and parental capability wellbeing. Longitudinal data are warranted to further validate our findings. Future studies may also explore the potential mediation role of perceived financial stress and work disruption in the relationship between child’s disease severity and parent’s capability wellbeing. Third, in the absence of Chinese-specific tariffs, we used UK tariffs to calculate ICECAP-A score. This may compromise the measure validity in Chinese population given the contextual differences. Chinese-specific tariffs that align with local social and cultural norm should be developed to assess the capability wellbeing more accurately in the Chinese context. Fourth, to standardize the assessment of disease severity across diverse conditions, we used dependence on assistive devices as a proxy measure, focusing on functional impact and support needs rather than impairment on affected systems. Moreover, due to the lack of a complete sampling frame of RD patients in China, the participants were recruited with non-probability sampling method. To maximize outreach, RDPOs conducted multiple rounds of patient engagement through various communication channels such as social platforms, websites, email newsletters, and patient support groups. In addition, RDPOs encouraged registered members to share recruitment information within their networks and engaged directly with patients by organizing virtual meetings and events to raise their awareness about the study and address any questions or concerns regarding participation. Despite these efforts, participants recruited through patient organizations may still have higher levels of self-awareness, social support, and access to disease-related knowledge and resources, compared to those who are not affiliated with such organizations. This limits the generalizability of our results, as rural or underserved areas with limited access to RDPOs may be prone to higher levels of distress and lower self-efficacy. This bias may have contributed to an overestimation of ICECAP-A scores. Future studies should consider incorporating broader recruitment strategies, such as collaboration with local healthcare providers and community organizations, to capture a more diverse and representative sample of the RD patient population. Additionally, as the fathers who participated in this study are more likely to be actively involved in caregiving responsibilities, the observed gender disparity may be underestimated. However, this sampling approach remains the most practical and feasible given the rarity and widespread distribution of RD patients across China. Considering the shared challenges faced by parents of pediatric patients, we believe that our findings have provided valuable implications with a degree of applicability to settings beyond China. Lastly, this study did not examine parents’ detailed demographics, health status, alternative caregiving obligations, support systems, utilization of government support and social services, or psychosocial aspects such as coping strategies. Mental health status of the parent, such as perceived stress, anxiety, and depression may be an important contributor or potentially moderate the relationship between child’s condition and parent’s wellbeing. Further research should include these variables to better understand capability wellbeing and its mechanisms. As our findings reflected Chinese gender roles, sociological analysis is needed to further explore their broader impact.

This study yields several key implications. First, the findings reveal the multifaceted burdens faced by parents caring for children with RDs, extending across caregiving, financial, and social realms. Through the lens of capability approach, our findings highlight the need for policies that address wellbeing beyond traditional healthcare. Second, interventions should adopt a family-centered approach that incorporates psychosocial support such as cognitive-behavioral therapy and tailored strategies. These measures should be targeted to help the parents, especially mothers, cope with disease-specific limitations and family-related stress. Establishing peer support groups among parents of children with RDs can improve access to disease-related information and provide social support. Empowerment programs should be designed for mothers to enhance self-esteem and self-efficacy, while fathers should be encouraged to participate more in caregiving, social events, and recreational activities. Third, continuous efforts are needed to improve diagnostic techniques and ensure access to healthcare for RDs, in order to help alleviate disease burdens for both pediatric patients and their parents. Fourth, while universal financial aids and drug pricing policies could effectively relieve parental burden, additional financial and social protections should be provided for families from lower socio-economic backgrounds. For example, government-subsidized respite care program could provide temporary caregiving support to reduce financial stress and minimize work interruption. To better support families caring for children with RDs, it is essential to implement flexible work arrangements, such as paid caregiving leave, remote work options, and flexible scheduling to accommodate healthcare and caregiving responsibilities. Additionally, raising societal awareness of RDs through public education campaigns and promoting peer support networks for caregivers could help reduce stigma and provide emotional and practical support to the affected families.

## Conclusions

Parents caring for children with rare diseases in China demonstrate impaired capability wellbeing, with particular challenges in areas of stability, achievement, and autonomy. There is an urgent need for more comprehensive, family-centered approaches in rare disease policies and services. To improve the overall wellbeing of families affected by rare diseases, it is important to expand support through psychosocial interventions, targeted workplace accommodations, and increased societal engagement.

## Supplementary Information

Supplementary Material 1.

## Data Availability

Individual-level information used for this study are not able to be shared by the authors. A request to receive data may be made to Peking Union Medical College Hospital and the Chinese University of Hong Kong. Upon reasonable request, the deidentified data can be made available to investigators whose proposed use of the data has been approved by an independent review committee. All enquiries should be sent to the corresponding author.

## Abbreviations

ANOVA: Analysis of variance
CAH: Congenital adrenal hyperplasia
CI: Confidence interval
EU: European Union
HRQoL: Health-related quality of life
ICECAP: ICEpop CAPability
ICECAP-A: ICEpop CAPability measure for adults
ICD-10: International Classification of Diseases, 10th Revision
IPAH: Idiopathic pulmonary arterial hypertension
mMOS-SSS: The Modified Medical Outcomes Study Social Support Survey
QoL: Quality of life
RDs: Rare diseases
SD: Standard deviation
UK: United Kingdom
