# Supplementary material for: Capability wellbeing of parents caring for children with rare diseases: a national cross-sectional study
Source: BMC Public Health. 2025 Nov 28;25:4190. doi: 10.1186/s12889-025-25452-8 (PMC12664264; doi:10.1186/s12889-025-25452-8)
Supplement: Supplementary file 1 — Supplementary Material 1. [file 12889_2025_25452_MOESM1_ESM.docx]

**Supplementary Materials.**


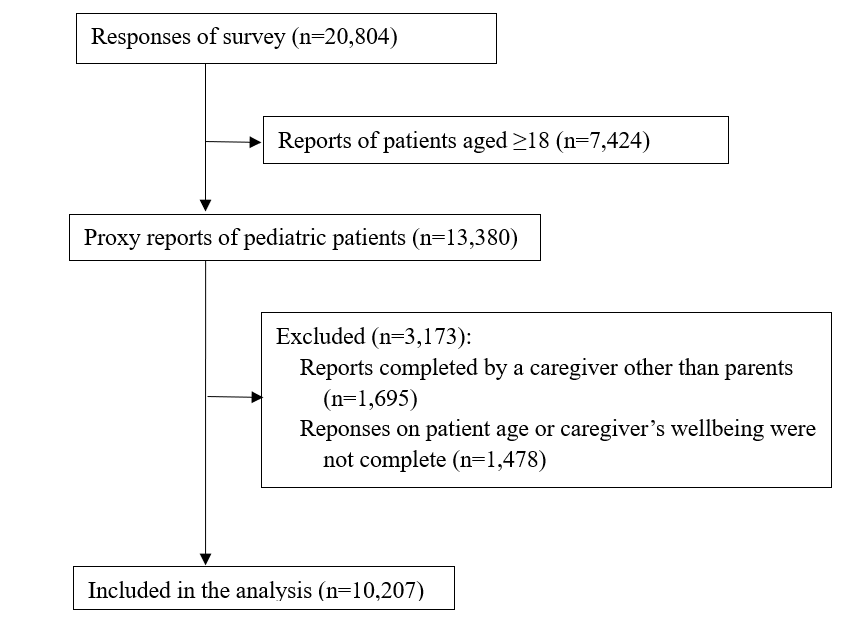


**Supplementary Figure 1.** Selection of participants included in the analysis.

**Supplementary Table 1.** Coefficient tariff of each item of ICEpop CAPability-Adult (ICECAP-A), by Flynn et al., 2015 ^1,2^

| **Item and response** | | **Tariff** |
| --- | --- | --- |
| **1. Feeling settled and secure** | |  |
| Level 4 | I am able to feel settled and secure in **all** areas of my life | 0.2221 |
| Level 3 | I am able to feel settled and secure in **many** areas of my life | 0.1915 |
| Level 2 | I am able to feel settled and secure in **a few** areas of my life | 0.1013 |
| Level 1 | I am **unable** to feel settled and secure in **any** areas of my life | -0.0008 |
| **2. Love, friendship and support** | | |
| Level 4 | I can have **a lot** of love, friendship and support | 0.2276 |
| Level 3 | I can have **quite a lot** of love, friendship and support | 0.1890 |
| Level 2 | I can have **a little** love, friendship and support | 0.0964 |
| Level 1 | I **cannot** have any love, friendship and support | -0.0239 |
| **3. Being independent** | | |
| Level 4 | I am able to be **completely** independent | 0.1881 |
| Level 3 | I am able to be independent in **many** things | 0.1560 |
| Level 2 | I am able to be independent in **a few** things | 0.0836 |
| Level 1 | I am **unable** to be at all independent | 0.0063 |
| **4. Achievement and progress** | | |
| Level 4 | I can achieve and progress in **all** aspects of my life | 0.1811 |
| Level 3 | I can achieve and progress in **many** aspects of my life | 0.1588 |
| Level 2 | I can achieve and progress in **a few** aspects of my life | 0.0909 |
| Level 1 | I **cannot** achieve and progress in **any** aspects of my life | 0.0210 |
| **5. Enjoyment and pleasure** | | |
| Level 4 | I can have **a lot** of enjoyment and pleasure | 0.1811 |
| Level 3 | I can have **quite a lot** of enjoyment and pleasure | 0.1540 |
| Level 2 | I can have **a little** enjoyment and pleasure | 0.0693 |
| Level 1 | I **cannot** have any enjoyment and pleasure | -0.0026 |

Level 4 indicate full capability; Level 1 indicates no capability. The total score of ICECAP-A is calculated by summing the values across the individual attributes. ICECAP-A: ICEpop CAPability Instrument for Adults.

**Supplementary Table 2.** Number of pediatric patients and ICECAP-A score of parents by disease according to disease and ICD-10.

| Disease | Patient (n=10,207) | ICECAP-A score  of parents | | |
| --- | --- | --- | --- | --- |
|  |  | Overall | Mothers | Fathers |
| *Endocrine, nutritional and metabolic diseases* | *n (%)* | *Mean (SD)* | *Mean (SD)* | *Mean (SD)* |
| Phenylketonuria | 2523 (24.7%) | 0.65 (0.22) | 0.65 (0.22) | 0.65 (0.22) |
| Albinism | 281 (2.8%) | 0.75 (0.18) | 0.76 (0.17) | 0.75 (0.19) |
| Mucopolysaccharidosis (MPS) | 235 (2.3%) | 0.58 (0.23) | 0.60 (0.24) | 0.57 (0.22) |
| Tetrahydrobiopterin deficiency | 214 (2.1%) | 0.61 (0.24) | 0.61 (0.23) | 0.60 (0.25) |
| Hepatolenticular degeneration (HLD); Wilson disease | 99 (1.0%) | 0.66 (0.25) | 0.63 (0.24) | 0.68 (0.25) |
| Gaucher disease (GD) | 85 (0.8%) | 0.58 (0.23) | 0.56 (0.25) | 0.59 (0.22) |
| Niemann-Pick disease (NPC) | 63 (0.6%) | 0.60 (0.24) | 0.59 (0.23) | 0.61 (0.26) |
| Glycogen storage disease type II (pompe disease) | 38 (0.4%) | 0.61 (0.23) | 0.62 (0.24) | 0.60 (0.22) |
| Fabry disease | 20 (0.2%) | 0.63 (0.16) | 0.67 (0.17) | 0.59 (0.14) |
| Homozygous familial hypercholesterolemia (HoFH) | 10 (0.1%) | 0.56 (0.22) | 0.60 (0.26) | 0.47 (0.08) |
| Congenital adrenal hyperplasia (CAH) | 876 (8.6%) | 0.72 (0.20) | 0.72 (0.20) | 0.72 (0.20) |
| Kallmann syndrome (KS) | 2 (0.02%) | 0.58 (0.24) | 0.41 (NA) | 0.76 (NA) |
| *Diseases of the nervous system* |  |  |  |  |
| Duchenne Muscular Dystrophy (DMD) | 1270 (12.4%) | 0.58 (0.23) | 0.55 (0.24) | 0.58 (0.23) |
| Multiple sclerosis (MS) | 10 (0.1%) | 0.65 (0.23) | 0.61 (0.25) | 0.75 (0.21) |
| Neuromyelitis optica spectrum disorder (NMOSD) | 12 (0.1%) | 0.70 (0.24) | 0.65 (0.17) | 0.75 (0.30) |
| Myasthenia gravis (MG) | 210 (2.1%) | 0.72 (0.20) | 0.72 (0.19) | 0.71 (0.20) |
| Dravet syndrome (DS) | 344 (3.4%) | 0.53 (0.23) | 0.53 (0.24) | 0.52 (0.23) |
| Huntington's Disease (HD) | 5 (0.1%) | 0.52 (0.24) | 0.42 (0.43) | 0.58 (0.08) |
| Spinal muscular atrophy (SMA) | 859 (8.4%) | 0.59 (0.21) | 0.58 (0.20) | 0.59 (0.22) |
| *Congenital malformations, deformations and chromosomal abnormalities* | | |  |  |
| Tuberous sclerosis complex (TSC) | 880 (8.6%) | 0.62 (0.22) | 0.62 (0.23) | 0.62 (0.22) |
| Prader-Willi Syndrome (PWS) | 563 (5.5%) | 0.58 (0.22) | 0.59 (0.22) | 0.57 (0.22) |
| Inherited epidermolysis bullosa (EB) | 391 (3.8%) | 0.68 (0.21) | 0.68 (0.20) | 0.68 (0.21) |
| Marfan syndrome (MS) | 95 (0.9%) | 0.67 (0.21) | 0.69 (0.19) | 0.65 (0.23) |
| Osteogenesis imperfecta (OI) | 60 (0.6%) | 0.64 (0.21) | 0.59 (0.19) | 0.68 (0.23) |
| *Diseases of the blood and blood-forming organs and certain disorders involving the immune mechanism* | | |  |  |
| Hemophilia | 777 (7.6%) | 0.68 (0.21) | 0.71 (0.22) | 0.68 (0.21) |
| *Diseases of the musculoskeletal system and connective tissue* | |  |  |  |
| Systemic sclerosis (SSc) | 157 (1.5%) | 0.73 (0.2) | 0.72 (0.21) | 0.76 (0.19) |
| *Neoplasms* |  |  |  |  |
| Langerhans cell histiocytosis (LCH) | 123 (1.2%) | 0.70 (0.20) | 0.72 (0.20) | 0.69 (0.20) |
| *Diseases of the circulatory system* |  |  |  |  |
| Idiopathic pulmonary arterial hypertension (IPAH) | 5 (0.1%) | 0.48 (0.24) | 0.35 (0.02) | 0.57 (0.29) |

ICECAP-A: ICEpop CAPability Instrument for Adults. SD: standard deviation.

**Supplementary Table 2.** Associations between significant factors and attribute-specific tariff.

1. Mothers

| Variable | ICECAP-A attribute | | | | |
| --- | --- | --- | --- | --- | --- |
|  | Stability | Attachment | Autonomy | Achievement | Enjoyment |
| ***Child variables*** | *Coeff (95% CI)* | *Coeff (95% CI)* | *Coeff (95% CI)* | *Coeff (95% CI)* | *Coeff (95% CI)* |
| **Age** | 0.001 (0, 0.001)* | 0.001 (0, 0.001)* | —— | —— | —— |
| **Dependence on assistive devices** |  |  |  |  |  |
| None | Ref. | Ref. | Ref. | Ref. | Ref. |
| Moderate | -0.005 (-0.011, 0.001) | -0.005 (-0.01, 0)* | -0.011 (-0.015, -0.007)*** | -0.006 (-0.009, -0.002)** | -0.004 (-0.008, 0)* |
| Substantial | -0.007 (-0.013, 0)* | 0.001 (-0.004, 0.007) | -0.021 (-0.026, -0.016)*** | -0.013 (-0.017, -0.009)*** | -0.005 (-0.01, -0.001)* |
| **Active treatment** |  |  |  |  |  |
| Non-active | —— | Ref. | —— | Ref. | Ref. |
| Active | —— | 0.007 (0.003, 0.011)** | —— | 0.004 (0.001, 0.007)* | 0.003 (0, 0.007)* |
| **Disease category** |  |  |  |  |  |
| Endocrine, nutritional and metabolic diseases | Ref. | Ref. | Ref. | Ref. | Ref. |
| Diseases of the nervous system | -0.014 (-0.019, -0.008)*** | -0.011 (-0.016, -0.006)*** | -0.013 (-0.017, -0.009)*** | -0.013 (-0.017, -0.01)*** | -0.008 (-0.012, -0.005)*** |
| Congenital malformations, deformations | -0.006 (-0.012, -0.001)* | -0.002 (-0.007, 0.003) | -0.005 (-0.009, -0.001)* | -0.007 (-0.011, -0.003)*** | -0.004 (-0.008, 0)* |
| Others | 0.003 (-0.004, 0.01) | 0.007 (0.001, 0.014)* | -0.002 (-0.008, 0.003) | 0.001 (-0.003, 0.006) | 0 (-0.005, 0.004) |
| ***Parental variables*** |  |  |  |  |  |
| **Primary caregiver of the patient** |  |  |  |  |  |
| No | Ref. | —— | Ref. | —— | —— |
| Yes | 0.008 (0.004, 0.012)*** | —— | 0.005 (0.002, 0.008)** | —— | —— |
| **Educational attainment** |  |  |  |  |  |
| Up to lower secondary | Ref. | Ref. | Ref. | Ref. | —— |
| Upper secondary | 0.008 (0.002, 0.013)** | 0.005 (0, 0.01)* | 0.012 (0.008, 0.016)*** | 0.004 (0.001, 0.007)* | —— |
| Tertiary | 0.012 (0.006, 0.018)*** | 0.006 (0.001, 0.012)* | 0.013 (0.008, 0.018)*** | 0.005 (0.001, 0.009)* | —— |
| **Annual household income** |  |  |  |  |  |
| Low (<¥50,000) | Ref. | Ref. | Ref. | Ref. | Ref. |
| Middle (¥50,000-100,000) | 0.007 (0.002, 0.012)** | 0.006 (0.001, 0.01)* | 0.007 (0.003, 0.01)*** | 0.004 (0.001, 0.008)** | 0.005 (0.002, 0.008)** |
| High (>¥100,000) | 0.012 (0.005, 0.018)*** | 0.009 (0.004, 0.015)** | 0.015 (0.01, 0.02)*** | 0.006 (0.002, 0.01)** | 0.006 (0.002, 0.01)** |
| **Social support** | 0.001 (0.001, 0.001)*** | 0.001 (0.001, 0.001)*** | 0.001 (0.001, 0.001)*** | 0.001 (0, 0.001)*** | 0.001 (0, 0.001)*** |
| **Perceived financial burden of the disease** | |  |  |  |  |
| None to moderate | Ref. | Ref. | Ref. | Ref. | Ref. |
| Severe | -0.017 (-0.022, -0.013)*** | -0.011 (-0.015, -0.007)*** | -0.012 (-0.016, -0.009)*** | -0.013 (-0.016, -0.01)*** | -0.015 (-0.018, -0.012)*** |
| **Perceived work disruption due to the disease** | |  |  |  |  |
| Never to sometimes | Ref. | Ref. | Ref. | Ref. | Ref. |
| Often or always | -0.026 (-0.031, -0.022)*** | -0.02 (-0.024, -0.016)*** | -0.013 (-0.016, -0.01)*** | -0.011 (-0.014, -0.008)*** | -0.015 (-0.018, -0.012)*** |

1. Fathers

| Variable | ICECAP-A attribute | | | | |
| --- | --- | --- | --- | --- | --- |
|  | Stability | Attachment | Autonomy | Achievement | Enjoyment |
| ***Child variables*** | *Coeff (95% CI)* | *Coeff (95% CI)* | *Coeff (95% CI)* | *Coeff (95% CI)* | *Coeff (95% CI)* |
| **Dependence on assistive devices** |  |  |  |  |  |
| None | Ref. | Ref. | Ref. | Ref. | Ref. |
| Moderate | -0.01 (-0.017, -0.003)** | -0.009 (-0.015, -0.002)** | -0.01 (-0.015, -0.005)*** | -0.008 (-0.012, -0.003)** | -0.005 (-0.01, 0) |
| Substantial | -0.013 (-0.021, -0.005)** | -0.005 (-0.013, 0.002) | -0.019 (-0.024, -0.014)*** | -0.017 (-0.022, -0.011)*** | -0.009 (-0.015, -0.003)** |
| **Active treatment** |  |  |  |  |  |
| Non-active | —— | —— | Ref. | —— | —— |
| Active | —— | —— | 0.01 (0.005, 0.014)*** | —— | —— |
| **Diagnostic delay** |  |  |  |  |  |
| <1 year | —— | —— | —— | Ref. | —— |
| 1-2 years | —— | —— | —— | -0.005 (-0.009, 0)* | —— |
| ≥3 years | —— | —— | —— | 0 (-0.008, 0.007) | —— |
| **Disease category** |  |  |  |  |  |
| Endocrine, nutritional and metabolic diseases | Ref. | Ref. | —— | Ref. | Ref. |
| Diseases of the nervous system | -0.003 (-0.011, 0.004) | -0.006 (-0.013, 0) | —— | -0.008 (-0.013, -0.003)** | -0.01 (-0.015, -0.005)*** |
| Congenital malformations, deformations | 0.007 (0, 0.015) | 0.011 (0.004, 0.018)** | —— | -0.001 (-0.006, 0.005) | 0.002 (-0.004, 0.007) |
| Others | 0.013 (0.004, 0.022)** | 0.017 (0.009, 0.025)*** | —— | 0.005 (-0.001, 0.011) | 0.006 (-0.001, 0.012) |
| ***Parental variables*** |  |  |  |  |  |
| **Educational attainment** |  |  |  |  |  |
| Up to lower secondary | Ref. | Ref. | Ref. | Ref. | Ref. |
| Upper secondary | 0.012 (0.005, 0.018)*** | 0.007 (0.001, 0.013)* | 0.013 (0.008, 0.017)*** | 0.006 (0.002, 0.011)** | 0.003 (-0.002, 0.007) |
| Tertiary | 0.013 (0.006, 0.02)*** | 0.009 (0.002, 0.017)* | 0.015 (0.009, 0.021)*** | 0.009 (0.003, 0.014)** | 0.009 (0.003, 0.015)** |
| **Employment** |  |  |  |  |  |
| Unemployed | —— | —— | Ref. | —— | Ref. |
| Employed |  | —— | 0.008 (0.002, 0.014)** | —— | 0.007 (0.001, 0.013)* |
| **Annual household income** |  |  |  |  |  |
| Low (<¥50,000) | —— | Ref. | Ref. | Ref. | Ref. |
| Middle (¥50,000-100,000) | —— | 0.007 (0.001, 0.013)* | 0.002 (-0.003, 0.007) | 0.004 (0, 0.009) | 0.01 (0.005, 0.014)*** |
| High (>¥100,000) | —— | 0.01 (0.002, 0.018)* | 0.008 (0.001, 0.014)* | 0.008 (0.002, 0.014)** | 0.01 (0.003, 0.016)** |
| **Social support** | 0.001 (0.001, 0.001)*** | 0.001 (0.001, 0.001)*** | —— | 0.001 (0, 0.001)*** | 0.001 (0, 0.001)*** |
| **Perceived financial burden of the disease** |  |  |  |  |  |
| None to moderate | Ref. | Ref. | Ref. | Ref. | Ref. |
| Severe | -0.023 (-0.03, -0.017)*** | -0.013 (-0.019, -0.007)*** | -0.012 (-0.017, -0.008)*** | -0.01 (-0.015, -0.006)*** | -0.011 (-0.015, -0.007)*** |
| **Perceived work disruption due to the disease** | |  |  |  |  |
| Never to sometimes | Ref. | Ref. | Ref. | Ref. | Ref. |
| Often or always | -0.025 (-0.031, -0.019)*** | -0.013 (-0.019, -0.008)*** | -0.018 (-0.022, -0.013)*** | -0.014 (-0.018, -0.009)*** | -0.02 (-0.025, -0.016)*** |

*p<0.05, **p<0.01, ***p<0.001, 1CNY¥=0.14USD$ ICECAP-A: ICEpop CAPability Instrument for Adults; CI: confidence interval; Ref: reference group.

References:

1. Flynn TN, Huynh E, Peters TJ, Al-Janabi H, Clemens S, Moody A, et al. Scoring the Icecap-a capability instrument. Estimation of a UK general population tariff. Health Econ. 2015 Mar;24(3):258–69.

2. Al-Janabi H, N Flynn T, Coast J. Development of a self-report measure of capability wellbeing for adults: the ICECAP-A. Qual Life Res. 2012;21(1):167–76.
